# Clinical Insights and Diagnostic Challenges of Pulmonary Lophomoniasis in a Sarcoidosis Patient: A Case Report

**DOI:** 10.1155/crpu/8752504

**Published:** 2026-04-28

**Authors:** Mohammad Hadi Tajik Jalayeri, Narges Lashkarbolouk, Mahdi Mazandarani, Mohaddeseh Dankoub

**Affiliations:** ^1^ Clinical Research Development Unit (CRDU), Sayyad Shirazi Hospital, Golestan University of Medical Sciences, Gorgan, Iran, goums.ac.ir; ^2^ Endocrinology and Metabolism Research Center, Tehran University of Medical Sciences, Tehran, Iran, tums.ac.ir

**Keywords:** *Lophomonas*, *Lophomonas blattarum*, parasite infection, pneumonia, sarcoidosis

## Abstract

**Introduction:**

In recent years, reports of patients with lophomoniasis infection have increased. This upward trend has been especially evident among patients with underlying conditions or those undergoing immunosuppressive therapy.

**Case Presentation:**

We present a 38‐year‐old Fars female patient of Fars ethnicity with a 2‐year medical history of sarcoidosis who was investigated due to persistent respiratory symptoms that were unresponsive to outpatient treatment. The patient was receiving immunosuppressive medication for her condition. During hospitalization, computed tomography of the lungs revealed the presence of cystic bronchiectasis in the lingula and linear atelectasis. Bronchoscopy was performed, and a bronchoalveolar lavage (BAL) sample was taken. Microscopic analysis of the sample showed the presence of *Lophomonas* spp.

**Conclusion:**

Sarcoidosis, an immunodeficiency‐related inflammatory disease, may increase susceptibility to parasitic infections. Pulmonary lophomoniasis, mostly presenting with nonspecific symptoms similar to other pneumonias, can lead to delayed diagnosis and poor outcomes. Physicians should consider lophomoniasis in immunocompromised patients due to differences in its nature, diagnostic approach, and treatment.

## 1. Introduction

Sarcoidosis is defined as a systemic disease of unknown etiology identified by the presence of noncaseating, nonnecrotizing epithelioid granulomas [1]. It exhibits a spectrum of clinical presentations and outcomes, ranging from spontaneous remission to the progression of severe respiratory impairment [2]. The condition typically onsets in adults under 50, with approximately 70% of cases emerging between the ages of 25 and 40, and a secondary peak of incidence observed in women over 50 years old [1]. The global incidence ranges from approximately 2 to 11 cases per 100,000 individuals annually, with about 90% of cases affecting the lungs, whereas smaller percentages involve the skin and eyes [2, 3].

The association between infection and sarcoidosis is significant. Certain studies suggest that infections may contribute to the development of sarcoidosis, as genetics accounts for only 31% of sarcoidosis cases, and environmental, infectious airborne contaminants, and occupational factors contribute up to 69%. At the same time, the nature of the disease and its treatments render patients susceptible to a range of infections, including opportunistic pathogens such as *Pneumocystis* pneumonia, invasive aspergillosis, and community‐acquired pneumonia (CAP) [4]. A 2006–2013 cohort study conducted by Rossides et al. found that sarcoidosis patients had a 1.8‐fold higher risk of serious infections and hospitalization than the general population, especially within 2 years of diagnosis [5]. Immunosuppressive therapy tripled the risk of serious infections, whereas untreated patients still experienced a 50% higher risk compared with controls. In addition, chronic immunosuppression, primarily due to the use of immunosuppressants such as corticosteroids, cytotoxic agents, and biologics, poses a significant infection risk by impairing immune cell functioning in these patients [4].

*Lophomonas blattarum* (*L. blattarum*) is a flagellated protozoan that resides in the hindgut of termites and cockroaches, classified under the *Lophomonas* suborder within the Hypermastigida order of the Parabasalia phylum [6]. The first documented case of pulmonary infection caused by this organism occurred in China in 1993 [7]. The parasite is excreted in cockroaches′ feces, where it forms cysts that persist in the environment. Although infections in humans are uncommon, they can occur in vulnerable individuals through inhalation of dust containing these cysts [6].

Pulmonary lophomoniasis is predominantly reported in tropical and subtropical regions, particularly in warmer climates with high cockroach populations [7]. According to a review study by Mewara et al. in 2024, they reported a prevalence of lophomoniasis of 17% based on a review of articles [8]. In addition, in the study by Nakhaei et al., the highest prevalence is in Asia (highest in Iran, China, and Turkey), followed by America, Africa, and Europe [7].

Infections from *Lophomonas* spp. are becoming more frequently recognized, particularly among immunocompromised individuals, such as those infected with human immunodeficiency virus (HIV), those on long‐term steroids or immunosuppressants, patients with malignancies, systemic illnesses, or chronic respiratory conditions [9]. Symptoms are often nonspecific, including fever, cough, sputum, hemoptysis, and shortness of breath, with radiological findings consistent with pneumonia‐related patterns, bronchiectasis, and pleural effusion [9, 10]. Diagnosis is made by examining morphological characteristics under light microscopy of fresh or stained samples, usually collected from sputum or bronchoalveolar lavages (BALs) during bronchoscopy [9, 10].

This case report describes a 38‐year‐old sarcoidosis patient who presented with a 4‐month history of respiratory symptoms unresponsive to outpatient treatment, ultimately diagnosed as a pulmonary *Lophomonas* spp. infection.

## 2. Case Presentation

A 38‐year‐old female patient of Fars ethnicity was referred to our pulmonary clinic at Sayyad Shirazi Hospital in Gorgan, Iran, presenting with persistent respiratory symptoms for about 4 months. Her clinical presentation included dyspnea and a nonpurulent cough, with no reports of fatigue, nocturnal sweating, hemoptysis, weight loss, anorexia, chills, or fever. The severity of her symptoms had worsened over the past 2 months, and an outpatient treatment was not effective. Her medical history revealed a diagnosis of sarcoidosis established 2 years prior. The diagnosis was based on presenting features such as blurred vision and conjunctival injection, which led to a diagnosis of uveitis, along with pulmonary lymphadenopathy and elevated angiotensin‐converting enzyme (ACE) levels, and her diagnosis was confirmed by a rheumatologist.

In her physical examination, vital signs were stable, and oxygen saturation was 97% without supplemental oxygen. Auscultation revealed respiratory crackles in both lungs, whereas heart sounds (S1 and S2) were found to be normal. Abdominal and pelvic examinations indicated no significant abnormalities, and there were no indicators of splenomegaly, hepatomegaly, or lymphadenopathy. Prednisolone 5 mg twice daily and methotrexate (MTX) 7.5 mg weekly were in her medication history, and her symptoms did not improve by increasing the prednisolone dosage.

Laboratory results revealed anemia (Hemoglobin 11), an erythrocyte sedimentation rate (ESR) of 45 mm/s, and negative C‐reactive protein (CRP) levels. White blood count was 12.7 × 10 3/*μ*L (neutrophil 65%), eosinophilia (10%), and platelet level was 236,000/*μ*L. Furthermore, given the ongoing COVID‐19 pandemic, a SARS‐CoV‐2 polymerase chain reaction (PCR) test was conducted, yielding a negative result. An investigation for fungal infection using a serum galactomannan test was also carried out, which returned negative.

A cardiology consultation was requested for the patient. The electrocardiographic evaluation indicated normal left and right ventricular function, with an ejection fraction ranging from 55% with no evidence of pericardial effusion.

**Figure 1:**
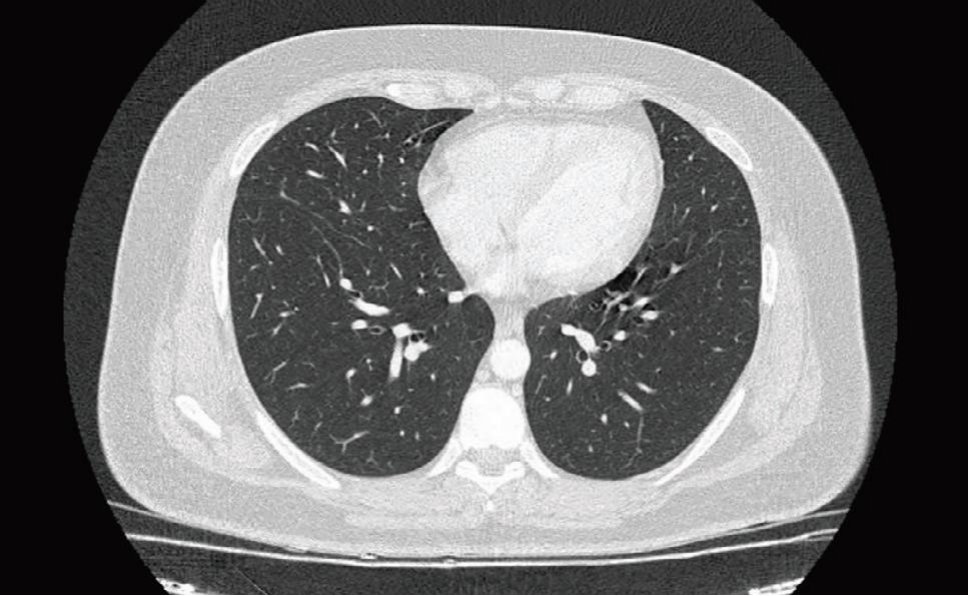
A lung computed tomography scan demonstrated cystic bronchiectasis in the lingula and linear atelectasis in the left lung.

**Figure 2:**
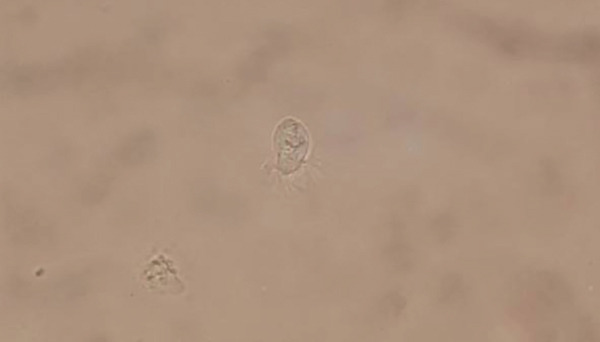
Direct microscopic examination showed a live, oval, and flagellated *Lophomonas* spp. protozoan in BAL sample of the patient.

Spiral CT of the lungs showed cystic bronchiectasis in the lingula and linear atelectasis in the left lung. No pleural effusion was observed (Figure 1). Fiber optic bronchoscopy was performed due to these findings, and BAL was collected to assess for BK, *Lophomonas*, bacterial, and fungal infections. BAL analysis was negative for fungal and bacterial infections. Microscopic examination of the BAL sample revealed multiple live, oval, flagellated *Lophomonas* spp. protozoa (Figure 2).

Consequently, the patient was diagnosed with an active pulmonary *Lophomonas* infection based on the positive BAL findings. Treatment with metronidazole 500 mg every 8 h was initiated. After 2 weeks of treatment, her condition improved, and she was subsequently discharged with instructions for oral medication, reporting overall good health and improved clinical symptoms. In follow‐up, after 3 months of treatment, there was no complaint of respiratory symptoms.

## 3. Discussion

Sarcoidosis is a multiorgan disease of unknown etiology characterized by nonnecrotizing granulomas in various organs. Although the cause remains unknown, the mechanisms of granuloma formation, including genetic susceptibility and environmental factors, are increasingly understood [11]. Common respiratory manifestations, such as cough (53%), dyspnea (51%), and chest pain (23%), are present in 30%–53% of patients. Chronic dyspnea is most common in patients with delayed diagnosis, including 10% of patients who develop sarcoidosis‐related lung fibrosis. Bilateral perihilar lymphadenopathy, frequently mediastinal, and perilymphatic pulmonary nodules, predominantly in the upper lobes, are typical imaging findings [1].

Sarcoidosis patients mostly experienced respiratory infections, followed by urinary tract infections, sepsis, pyelonephritis, erysipelas, gastroenteritis, and colitis [11]. Moreover, they are at risk for opportunistic infections, with glucocorticoid treatment being a major risk factor for them [11]. As mentioned in the studies, common opportunistic infections include aspergillosis, mycobacterial infection, cryptococcosis, and progressive multifocal leukoencephalopathy. Contrarily, a history of tuberculosis (TB) is associated with an eightfold increased risk of sarcoidosis, and newly diagnosed patients are at higher risk for TB. Histoplasmosis and *Cutibacterium acnes* (*C. acnes*) have also been linked to disease pathogenesis and should be considered in differential diagnoses [4, 11].

Lophomoniasis is a chronic respiratory infection affecting both the upper and lower respiratory tracts, causing fever, cough, sputum production, and pneumonia. It is primarily caused by *L. blattarum*, found in insects like cockroaches, with human infection occurring mainly through inhalation of cyst‐containing aerosols [12]. Although primarily detected in lower respiratory tract samples, it has also been found in sinusitis, bronchitis, allergic rhinitis, asthma, and, rarely, uterine and urinary tract infections. Consequently, clinical symptoms are variable and mostly nonspecific [8, 12]. As reported in a minireview of 58 patients with lophomoniasis, the most common symptoms were cough (70.6%), followed by fever (60.3%), expectoration (46.5%), and dyspnea (41.3%) [8]. Similar to our case, she was diagnosed with sarcoidosis 2 years ago and had persistent respiratory manifestations such as cough and dyspnea. Studies have shown that patients with a history of immunocompromised conditions are at risk of lophomoniasis infection [13]. Taheri et al. found one case of sarcoidosis among 34 patients with lophomoniasis in a cross‐sectional imaging study [14]. Berenji et al. reported a higher incidence of concurrent *Pneumocystis jirovecii* and lophomoniasis infections in elderly patients with respiratory infections [15]. Additionally, Shahkar et al. reported pulmonary lophomoniasis in a 16‐year‐old patient with autism, hydatid cysts, and TB [16].

The most common radiological findings in lophomoniasis include nodules with a tree‐in‐bud pattern, alveolar consolidation, bronchiectasis, and centrilobular nodules. Less frequent findings include peribronchial infiltrates, ground glass opacities, pleural effusions, and lung abscesses [15]. Laboratory results are also nonspecific, with leukocytosis, elevated inflammatory markers, and eosinophilia reported in 35% of patients [17, 18]. In line with our case, she had cystic bronchiectasis in the lingula and linear atelectasis in the left lung in her lung CT scan. In addition, anemia, elevated ESR, leukocytosis, and eosinophilia were detected on her laboratory evaluation.

Direct microscopic observation of motile trophozoites in BAL fluid or sputum samples remains the standard for diagnosis. However, differentiating it from ciliated epithelial cells is challenging due to morphological similarities, which can lead to misdiagnosis under light microscopy [9]. The epithelial cells of the lung are slightly slender with uniformly sized, consistently moving cilia and a basal terminal bar. *L. blattarum*, on the other hand, are oval‐ to pyriform‐shaped protozoa (20–60 *μ*m × 12–20 *μ*m) distinguished by a polar tuft of flagella (longer centrally and shorter peripherally) and a spherical nucleus [6]. There is limited data on the specificity and sensitivity of Giemsa, Papanicolaou, trichrome, and hematoxylin/eosin staining methods for detecting *Lophomonas* in patients. Moreover, there is a lack of standardized culture and serological tests for it [19]. Nevertheless, recent studies have shown promising results for detecting lophomonoasis in patients using PCR techniques [19]. In our case, we diagnosed lophomoniasis by direct microscopic examination of BAL, observing multiple live, oval, flagellated *Lophomonas* protozoa.

There is no standard guideline for treating *Lophomonas* infection. However, as stated in studies, lophomoniasis can be treated with metronidazole (adult: 500 mg every 8 h for 7–10 days; children: 5 mg/kg every 8 h for 7–10 days), or alternatively with tinidazole (500 mg orally twice daily for 5–6 days), or albendazole (400 mg orally daily for 5 days) [18]. Our patient was treated with a metronidazole protocol, and her respiratory symptoms resolved; she had no respiratory complaints during the 3‐month follow‐up.

## 4. Conclusion

Sarcoidosis is a chronic inflammatory condition associated with immunodeficiency, which can increase susceptibility to parasitic infections. Pulmonary lophomoniasis has nonspecific symptoms that can result in delayed diagnosis, increased morbidity, and higher healthcare costs. Clinicians should consider lophomoniasis in patients with persistent respiratory symptoms, as early diagnosis and treatment can improve outcomes.

## Author Contributions

M.H.T.J. and M.M. advised the case report study. N.L. and M.D. collected medical and health records of the patient. M.M. and N.L. wrote the first version of the manuscript, and all the authors of this study noted on previous versions.

## Funding

No funding was received for this manuscript.

## Disclosure

All authors comprehensively read and approved the final manuscript.

## Ethics Statement

Written informed consent was acquired from the patient for publication of this case report and any concomitant figures. A copy of the written consent is accessible for the purpose of the review by the editor in chief of the journal. The aim of this study was completely defined to the patient, and she was assured that her information would be kept private by the authors. This study was conducted in accordance with the principles of the Declaration of Helsinki.

## Consent

Written informed consent was acquired from the patient for publication of this case report and any concomitant figures. A copy of the written consent is accessible for the purpose of the review by the editor in chief of the journal.

## Conflicts of Interest

The authors declare no conflicts of interest.

## Data Availability

The data that support the findings of this study are available on request from the corresponding authors. The data are not publicly available due to privacy or ethical restrictions.
